# Riboregulators: Fine-Tuning Virulence in *Shigella*

**DOI:** 10.3389/fcimb.2016.00002

**Published:** 2016-01-27

**Authors:** Megan E. Fris, Erin R. Murphy

**Affiliations:** ^1^Department of Biological Science, Ohio UniversityAthens, OH, USA; ^2^Department of Biomedical Sciences, Heritage College of Osteopathic Medicine, Ohio UniversityAthens, OH, USA

**Keywords:** riboregulator, *Shigella*, sRNA, pathogenesis, environment, small RNA

## Abstract

Within the past several years, RNA-mediated regulation (ribo-regulation) has become increasingly recognized for its importance in controlling critical bacterial processes. Regulatory RNA molecules, or riboregulators, are perpetually responsive to changes within the micro-environment of a bacterium. Notably, several characterized riboregulators control virulence in pathogenic bacteria, as is the case for each riboregulator characterized to date in *Shigella*. The timing of virulence gene expression and the ability of the pathogen to adapt to rapidly changing environmental conditions is critical to the establishment and progression of infection by *Shigella* species; ribo-regulators mediate each of these important processes. This mini review will present the current state of knowledge regarding RNA-mediated regulation in *Shigella* by detailing the characterization and function of each identified riboregulator in these pathogens.

## Introduction

*Shigella* are bacterial pathogens highly adapted for colonizing the human gut, a process that is facilitated by their unique lifestyle. The bacteria are passed from host to host via the fecal oral route of transmission. After surviving the acidic environment of the stomach, *Shigella* species travel the length of the intestinal tract to the site of infection at the colonic epithelium (Jennison and Verma, 2003). Once at the colon, *Shigella* transverse the colonic epithelium by passage through M-cells and are subsequently presented to, and engulfed by, macrophages. Once inside the macrophage, *Shigella* species induces lysis of the phagocytic cell, releasing the bacteria to the basal-lateral surface of the epithelium. (Wassef et al., 1989) Next, *Shigella* invade human intestinal epithelial cells using a type three secretion system (TTSS) and begin to replicate within the eukaryotic cytoplasm. (Schroeder and Hilbi, 2008) Finally, the bacteria utilize host actin to spread from one eukaryotic cell to the next, a process that destroys intestinal epithelial cells thus contributing directly to the development of symptoms of a *Shigella* infection, namely bloody diarrhea (Jennison and Verma, 2003; Schroeder and Hilbi, 2008). The gene encoding any factor that directly or indirectly facilitates the ability of *Shigella* species to complete one or more process essential for pathogenesis must themselves be considered virulence-associated genes.

**Figure 1:**
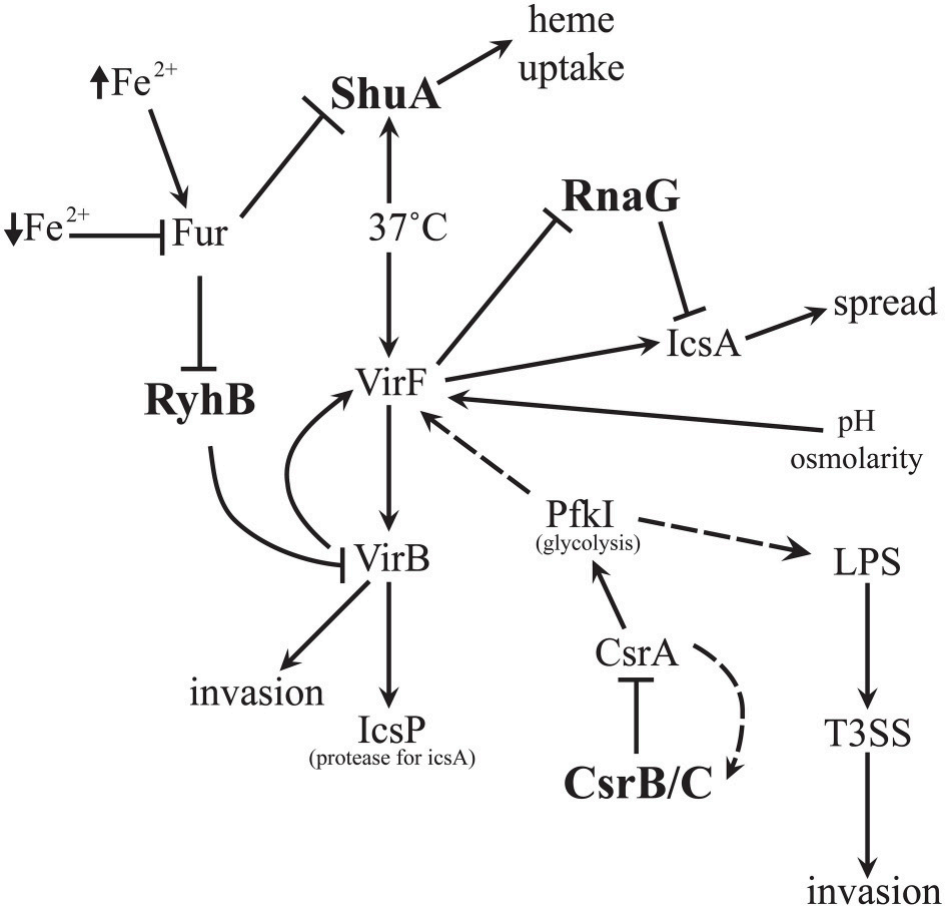
**sRNA Network Involved with ***Shigella*** Virulence**. All sRNAs which have been characterized in *Shigella* thus far are interconnected within the virulence network. RyhB inhibits VirB which can lead to inhibition of invasion. CsrB/C can inhibit CsrA which is needed for invasion. RnaG inhibits *icsA* transcription and limits spread. ShuA is important for heme uptake and survival of the pathogen during the infection process.

To establish and progress an efficient infection, *Shigella* species precisely regulate the expression of virulence-associated genes in response to specific environmental conditions encountered within the host; a collection of complex processes in which regulatory RNA molecules play critical, and ever increasingly recognized roles (Figure 1). Their ability to mediate a rapid, specific response makes riboregulators ideal molecules to mediate the regulation of virulence-associated gene expression in response to changes within a pathogen's micro-environment. Riboregulators characterized thus far in *Shigella* include several regulatory small RNAs (sRNAs) and one RNA thermometer (Murphy and Payne, 2007; Giangrossi et al., 2010; Gore and Payne, 2010; Africa et al., 2011; Tran et al., 2011; Broach et al., 2012; Kouse et al., 2013). Despite the fact that they function to regulate the expression of different target genes and that they utilize a variety of molecular mechanisms, all riboregulators described in *Shigella* to date share two important features; (1) they each respond, directly or indirectly, to changes in specific environmental conditions, and (2) they all are nestled within large regulatory networks that impact pathogenesis (Table 1 and Figure 1). This review will examine all characterized riboregulators in *Shigella*, with special emphasis placed on a discussion of how each was discovered, as well as their functions and impact on pathogenesis.

**Table 1:** **Summary of Riboregulators in ***Shigella*****.

**Riboregulator**	**Type**	**Environmental influence**	**Target**	**Virulence associated process**
CsrB/CsrC	Sequestering sRNAs	Carbon	CsrA	Invasion
RnaG	*cis*-encoded sRNA	Temperature	*icsA*	Spread
RyhB	*trans*-encoded sRNA	Iron	VirB	Invasion
5′ UTR ShuA	RNA thermometer	Iron and temperature	ShuA	Iron acquisition from heme

*Each riboregulator described in Shigella has also contributed to virulence of Shigella in some way. The sRNAs seem to effect invasion and spread while the RNA thermometer is important with iron acquisition*.

## CsrB and CsrC

Two sRNAs which are important regulatory molecules for carbon metabolism in *E. coli* are the carbon storage regulators CsrB and CsrC (Liu et al., 1997; Romeo, 1998; Weilbacher et al., 2003). CsrB and CsrC belong to a unique class of sRNAs which bind and sequester multiple copies of their target protein to inhibit its activity (Liu et al., 1997; Romeo, 1998; Weilbacher et al., 2003). In this case, CsrB and CsrC bind to and inhibit CsrA, a protein that promotes the production of other proteins necessary for glycolysis while inhibiting the production of proteins required for gluconeogenesis (Romeo et al., 1993; Sabnis et al., 1995; Yang et al., 1996; Liu et al., 1997; Weilbacher et al., 2003). CsrA also indirectly up-regulates the production of CsrB and CsrC through the UvrY/BarA two-component system, thus regulating its own activity (Gudapaty et al., 2001; Suzuki et al., 2002). Key genes involved with carbon regulation in *E. coli*, including those encoding CsrA, CsrB, and CsrC as well as UvrY and BarA, are conserved in *Shigella*.

Interestingly, researchers have demonstrated that CsrA activity is linked to virulence in S. flexneri (Gore and Payne, 2010). Specifically, a mutation in *csrA* inhibits the ability of S. flexneri to invade eukaryotic cells and to spread from one eukaryotic cell to the next within a monolayer (Gore and Payne, 2010). Additionally a mutation in *csrB* or *csrC* which, in turn, indirectly increases the amount of free CsrA, allows S. flexneri to invade slightly more effectively than the wild-type strain (Gore and Payne, 2010). When CsrB or CsrC are over-produced, CsrA activity is inhibited as expected, and S. flexneri is no longer able to invade host cells (Gore and Payne, 2010). CsrA is hypothesized to impact *Shigella* virulence by two mechanisms. By the first mechanism, CsrA facilitates the activity of phosphofructokinase A (PfkA), which in turn upregulates production the master virulence regulator in *Shigella*, VirF (Adler et al., 1989; Gore and Payne, 2010). By the second mechanism, CsrA and PfkA may impact glycosylation of LPS on the surface of *Shigella*. Changes in LPS structure, mediated by alterations in the degree of glycosylation, impact the exposure of the type III secretion system (T3SS) on the surface of *Shigella* (West et al., 2005). Given these observations, changes in the Csr regulatory pathway could influence exposure of the *Shigella* T3SS (Hong and Payne, 1997; West et al., 2005; Gore and Payne, 2010). Regardless of the underlying molecular mechanism, CsrA clearly impacts pathogenesis in *Shigella* (Gore and Payne, 2010). As RNA molecules that function to regulate CsrA activity, CsrB and CsrC are thus implicated in the control of *Shigella* virulence.

Although different in size, no unique functions for CsrC and CsrB have been found thus far in either *E. coli* or *Shigella*. Investigating CsrB and CsrC under a number of different environmental could reveal unique activity and/or production patterns for these regulators (Weilbacher et al., 2003). In general, the biological significance of sibling sRNAs with apparent functional redundancies remains unclear (Caswell et al., 2014). It has been suggested that the extremely short half-life of CsrB and CsrC contributes to the speed by which the sRNAs can regulate CsrA, and that CsrB and CsrC allow for fine-tuning of gene expression in response to changes of carbon sources (Romeo, 1998). The short half-life of CsrB and CsrC could also contribute to the rapid responses needed for the regulation of pathogenesis based on environment specific alterations in carbon source availability.

## RyhB

Another sRNA indirectly influenced by an environmental factor is RyhB. Originally discovered and characterized in *E. coli*, RyhB has been demonstrated to be an important regulator of iron metabolism (Massé and Gottesman, 2002). Bacteria need iron for survival, but too much iron can kill the organism, thus the production of iron uptake systems and iron storage systems are tightly regulated (Andrews et al., 2003). Studies in *E. coli* have demonstrated that RyhB plays an important role in maintaining the critical balance between the strict requirement and potential toxicity of iron (Massé and Gottesman, 2002; Andrews et al., 2003) Specifically, the production of RyhB itself is regulated in response to iron availability via the activity of the iron-responsive transcriptional regulator Fur (Bagg and Neilands, 1987; De Lorenzo et al., 1988; Hantke, 2001; Massé and Gottesman, 2002). Under conditions of high iron, Fur functions to inhibit RyhB production. Fur-dependent repression of RyhB production in turn relieves the RyhB-mediated repression of genes encoding various iron containing enzymes and iron storage proteins (Massé and Gottesman, 2002). RyhB is conserved between *E. coli* and *Shigella* where, as in *E. coli*, production of the sRNA is regulated by Fur, and activity of the sRNA impacts the expression of several targets conserved between the two genus (Murphy and Payne, 2007).

In addition to the role that RyhB plays in iron metabolism, RyhB has been implicated in the regulation of virulence-associated gene expression in *S. dysenteriae* (Murphy and Payne, 2007; Africa et al., 2011; Broach et al., 2012). Specifically, RyhB inhibits the transcription of *virB*, a gene encoding a protein that functions to promote the expression of several virulence-associated genes in *Shigella*. (Adler et al., 1989; Beloin et al., 2002; Murphy and Payne, 2007; Africa et al., 2011; Kane and Dorman, 2012). Although the exact molecular mechanism underlying RyhB-dependent inhibition of *virB* transcription remains unknown, complementarity between the template DNA strand within the *virB* open reading frame and RyhB exists, and is required for the observed regulation; data that suggests a novel regulatory mechanism may be at play (Broach et al., 2012).

RyhB allows for iron responsive regulation of the *Shigella* virulence cascade. In the relatively iron-rich environment of the human gut, Fur is likely active and functioning to repress the production of RyhB. With decreased production of RyhB, VirB production proceeds and the protein functions to promote the expression of several virulence-associated genes including *icsP* (Wing et al., 2004; Castellanos et al., 2009; Broach et al., 2012). IcsP protease limits IcsA (a protein required to polymerize the actin tail used by *Shigella* to spread from one eukaryotic cell to the next) from being produced prior to invasion into the host cell (Makino et al., 1986; Bernardini et al., 1989; Lett et al., 1989; Goldberg and Theriot, 1995; Egile et al., 1997; Shere et al., 1997; Steinhauer et al., 1999; Wing et al., 2005; Africa et al., 2011). Once *Shigella* enter the host cell, iron conditions become limiting, and as a result Fur-mediated repression of RyhB production is relieved. Once produced in the low iron environment RyhB functions to represses *virB* expression, which in turn limits IcsP production (Wing et al., 2004; Africa et al., 2011). Decreased IcsP production results in increased activity of IcsA which in turn facilitates host actin polymerization and cell to cell spreading by the bacterium (Makino et al., 1986; Bernardini et al., 1989; Lett et al., 1989; Goldberg et al., 1993; Goldberg and Theriot, 1995).

Inhibition of *virB* transcription is not the only way by which RyhB influences pathogenesis in *Shigella*. In addition to its role in modulating VirB production, RyhB also indirectly regulates the expression of genes encoding factors required for acid resistance, an essential aspect of infection initiation by this pathogen (Oglesby et al., 2005).

Evolutionarily, as an sRNA RyhB is likely a more adapt regulator than a protein would be. To compensate for small changes in iron availability within the human host, the synthesis of RyhB can quickly be inhibited by Fur. RyhB can also become a fully active regulator after only transcription, giving it an edge over a protein regulator which would require more energy and time to synthesize (Beisel and Storz, 2010). Its fundamental features as an iron-regulated ribo-regulator allow for quick, efficient modulation of target gene expression by RyhB in response to the subtle changes of environmental iron availability experienced by the pathogen throughout the course of a natural infection.

## RnaG

RnaG is unique among *Shigella* sRNA in that, unlike the others, it was first identified and characterized in *Shigella*, and it is encoded on the large virulence plasmid. Similar to other *Shigella* sRNAs however, is the fact that production of RnaG is regulated in response to a specific environmental cue and that once produced, it functions to impact pathogenesis. Specifically, RnaG production is indirectly controlled in response to environmental temperature, and once produced functions to regulate the transcription of *icsA*, a virulence-associated gene required for actin-based motility of *Shigella* species (Bernardini et al., 1989; Giangrossi et al., 2010; Tran et al., 2011). Two coordinated mechanisms allow RnaG to mediate transcriptional control of *icsA*. First, *rnaG* and *icsA* have convergent promoters in close proximity to each other (Giangrossi et al., 2010). As such, activity of the *rnaG* promoter results in decreased activity of the *icsA* promoter through promoter interference (Giangrossi et al., 2010). Second, as a result of their over-lapping arrangement, and thus nucleic acid complementarity, RnaG can bind directly to the *icsA* transcript via kissing complexes, alter the structure of the growing transcript, and lead to early transcriptional termination (Giangrossi et al., 2010). Through these two, non-mutually exclusive molecular mechanisms, transcription of the important virulence factor IcsA is inhibited by RnaG, thus directly implicating this small RNA in controlling *Shigella* pathogenesis.

RnaG is likely produced during the time *Shigella* first enters the host until the pathogen reaches the site of infection. During this time, RnaG would inhibit premature expression of *icsA*, thus preventing the production of proteins required for host actin polymerization (Giangrossi et al., 2010). At 37°C (the environmental temperature within the host), VirF is produced and functions to promote the transcription of *icsA*, thus inhibiting that of *rnaG* (Tran et al., 2011). During the initial stages of infection, however, it is possible that VirF levels are not high enough to induce *icsA* transcription to levels required for efficient actin polymerization and, due to specific environmental factors such as pH and osmolarity, may not be high enough until *Shigella* reaches the colonic epithelium (Porter and Dorman, 1994; Nakayama and Watanabe, 1995; Kane and Dorman, 2012). In this case, RnaG production during these initial stages of infection would inhibit aberrant *icsA* expression between the time that *Shigella* first enters the host and when the pathogen reaches the site of infection. Such temporal timing would prevent premature production of IcsA and possibly damper any immune system alarms which may be set off in the presence of the protein.

## ShuA

The final *Shigella* ribo-regulator is an RNA thermometer located within the 5' untranslated region (UTR) of *S. dysenteriae shuA* (*Shigella* heme uptake), a gene encoding an outer-membrane heme receptor that is essential for the utilization of heme as a source of nutrient iron by the pathogen (Mills and Payne, 1995, 1997). RNA thermometers function to modulate translation efficiency from the transcript on which they are housed by the formation of an inhibitory structure(s) that physically blocks binding of the ribosome to the transcript at non-permissive (low) temperatures (Kortmann and Narberhaus, 2012). As the environmental temperature rises the inhibitory structure is destabilized, the ribosomal binding site is exposed and translation of the regulated gene proceeds. The *shuA* RNA thermometer represents the first RNA thermometer characterized in any *Shigella* species (Kouse et al., 2013). Although identified initially in *Shigella*, an identical regulator functions to control expression of the orthologous gene *chuA* in pathogenic *E. coli* where this gene product is a virulence determinant (Wyckoff et al., 1998; Hoffmann et al., 2001; Torres et al., 2001; Okeke et al., 2004). Transcription of *shuA* is subject to iron-dependent regulation by the protein Fur while translation from the *shuA* transcript is subject to temperature-dependent regulation by the activity of the *cis*-encoded RNA thermometer (Mills and Payne, 1995, 1997; Kouse et al., 2013). It is important to note, that for bacterial pathogens increased environmental temperature can act as an important signal that the organism has entered the host, the environment where production of virulence-associated factors will provide the most benefit.

Only under particular environmental conditions will ShuA be efficiently produced. Under conditions where iron is abundant, *shuA* transcription will be repressed by the activity of Fur, regardless of environmental temperature. Conditions where iron is depleted, but the environmental temperature is relatively low, the FourU RNA thermometer will inhibit translation of *shuA*. Only in iron-limiting and at temperatures corresponding to that within the human body (37°C), will ShuA be produced (Kouse et al., 2013). The transcriptional and translation regulation mediated by Fur and the *shuA* RNA thermometer, respectively, function together to ensure maximal production of ShuA under conditions of poor iron availability and increased temperature, precisely the condition encountered within the human body; the only environment in which *Shigella* will encounter heme as a potential source of essential nutrient iron.

## Discussion

The riboregulators in *Shigella*, described in this review, all respond (directly or indirectly) to environmental changes, and all of them function within larger regulatory networks to influence pathogenesis of these species. CsrB/CsrC are regulated in response to carbon availability, RnaG is regulated in response to temperature, RyhB is regulated in response to iron availability, and finally the activity of the *shuA* RNA thermometer is regulated in response to temperature. Importantly, every *Shigella* ribo-regulator characterized to date functions to influence the production of factors involved in one or more processes required for pathogenesis, and thus must themselves be considered virulence determinants. This observation raises the question, why are some virulence-associated processes in *Shigella* controlled by protein-based regulation while others are controlled, at least in part, by the activity of riboregulators? In all sRNA found in *Shigella* thus far, proteins (VirF, H-NS, Fur, and UvrY) seem to be the initial regulator controlling a given step of a specific virulence-associated process. In each case, the ribo-regulator functions to modulate a specific virulence-associated activity for some duration of time, and then due to an environmental trigger, quickly switches off and allows *Shigella* to proceed to the next stage of pathogenesis (Figure 1). Perhaps *Shigella* evolved to favor riboregulators over protein regulators in conditions under which rapid specific changes to the production of one or just a few genes would be more beneficial to the organism than turning on/off an entire large regulon. Perhaps, processes required for the initial induction of pathogenesis in *Shigella* is controlled by protein regulators rather than riboregulators because quick reactions to false positive signals for pathogenesis could be detrimental to the survival of the bacteria, while a lag in protein regulation may temper those signals, thus reducing the frequency of such detrimental events. (Beisel and Storz, 2010).

More research needs to be done on riboregulators in *Shigella* to fully understand their functions and roles in virulence regulation (Storz et al., 2011). Such studies are likely to be fueled by genomic-based analyses that suggest the presence of additional ribo-regulators in *Shigella*, regulators for which function(s) have not yet been elucidated (Peng et al., 2011; Skippington and Ragan, 2012). Additionally, many putative sRNAs should be examined to see if they have small proteins missed by predictor programs (Storz et al., 2014). Lastly, once sRNAs and riboregulators are fully understood, it is possible that their function could be targeted as novel treatments for shigellosis.

## Author contributions

Wrote the article: MF and EM. Edited the article: MF and EM. Created the figure: MF. Created the table: MF.

## Funding

Studies in the Murphy lab have been supported by the National Institutes of Health, the American Heart Association, Ohio University, and Ohio University College of Osteopathic Medicine.

### Conflict of interest statement

The authors declare that the research was conducted in the absence of any commercial or financial relationships that could be construed as a potential conflict of interest.
